# Structure, Conformations, and Diffusion in PDMS/Silica Nanocomposites via Atomistic MD Simulations

**DOI:** 10.1021/acs.macromol.5c01745

**Published:** 2025-11-25

**Authors:** Argyrios V. Karatrantos, Nigel Clarke, Lyazid Bouhala, Clement Mugemana, Martin Kröger

**Affiliations:** † Materials Research and Technology, 87145 Luxembourg Institute of Science and Technology, 5, Avenue des Hauts-Fourneaux, L-4362 Esch-sur-Alzette, Luxembourg; ‡ School of Mathematical and Physical Sciences, 7315 University of Sheffield, 5, Sheffield S3 7RH, U.K.; § Computational Polymer Physics, Department of Materials, ETH Zurich, 8093 Zurich, Switzerland; ∥ Magnetism and Interface Physics, Department of Materials, Leopold-Ruzicka-Weg 4, ETH Zurich, 8093 Zurich, Switzerland

## Abstract

Poly­(dimethylsiloxane) (PDMS)–silica nanocomposites have attracted increasing attention due to their outstanding inherent properties, such as mechanical strength, self-healing, and superhydrophobicity. In this work, we explore the structure, conformations, and diffusion of neutral (nonionic) and ionic PDMS melts confined between nanosilica surfaces, using atomistic molecular dynamics, to provide a nanoscale insight into the interface and interphase, which play a crucial role in the design of novel nanocomposites. We investigate the effect of hydrogen bonding and ionic interactions, together with temperature, chain charge density, electrostatic strength, and charge localization, on the structure and dynamics of the PDMS chains. The chain charge density altered the structure of PDMS chains near the ionic functionalized nanosilica surface. In particular, it is observed that functionalized ionic chain-end PDMS obtains the largest dimensions, while a chain charge density of 10% lead to the contraction of PDMS chains in comparison with neutral PDMS chains. In addition, the ionic functionalization of the PDMS and nanosilica surface decrease the chain dynamics compared with the van der Waals dispersion and hydrogen bonding interactions. Hydrogen bonds between silanols and oxygen of PDMS are affected by the molecular weight of the neutral PDMS chains. Neutral short PDMS chains appear to have faster diffusion and interfacial dynamics, and the neutral long or ionic PDMS chains show a subdiffusive behavior. Charge localization or electrostatic strength has a substantial effect on ionic PDMS chains’ structure, conformations, dynamics, and adhesion, while temperature has a negligible effect for neutral PDMS chains.

## Graphical abstract

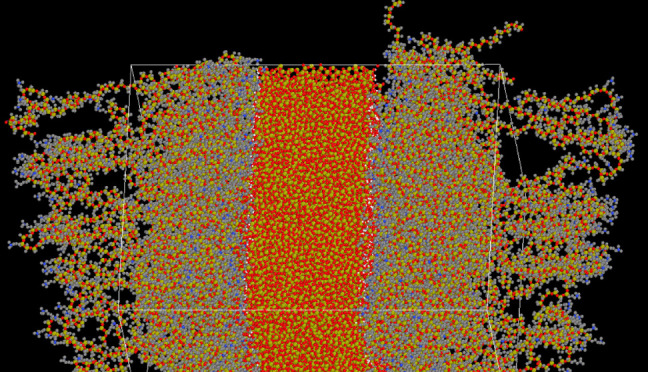

## 1 Introduction

Polydimethylsiloxane (PDMS) is the most widely explored and utilized polysiloxane due to its extremely low glass transition (*T*_g_ = −125 °C), nontoxicity, excellent thermal stability, high gas permeability, fine optical transparency, oxidative stability, UV resistance, and good biocompatibility.^1,2^ Recently, there have been experimental efforts in ionic PDMS/nanosilica composites, where PDMS was functionalized with tertiary amine N^+^(CH_3_)_3_ groups, either at their chain ends (telechelic) or randomly grafted along the backbone (random copolymer),^3^ and an electrostatic interaction took place with a sulfonate functionalization (SO_3_^–^) on the nanosilica surface. The use of reversible ionic interactions at the interface promoted a nanosilica dispersion state and resulted in a PDMS/silica nanocomposite with self-healing capability.^3^ Klonos et al.^4–7^ studied the effects on the structure,^8^ molecular mobility, and interfacial polymer–nanosilica particles by grafting small polydimethylsiloxane (PDMS) chains, via siloxane bond breaking^9^ for different polymer molecular weights (*M*_w_). Their results suggested the formation of large-height interfacial PDMS loops, which eliminated the ability for cooperative motions due to interfacial chain entanglements. The adhesion of PDMS can be affected by temperature, *M*_w_, and the polarity of the terminal group, as was observed on neutral PDMS/nanosilica interfaces.^10^ Patel et al. used Fourier transform infrared (FTIR) spectroscopy to examine the interaction of different chain-end groups—hydroxyl, methyl, and vinyl—on functionalized poly­(dimethylsiloxane) (PDMS) with nanosilica, finding that hydroxyls exhibited the strongest affinity.^11^ Gong et al. reported on the relationship between bound-layer thickness and nanosilica particle size in PDMS–silica nanocomposites.^12^ Arrighi et al. used quasielastic neutron scattering (QENS) to study the dynamics of poly­(dimethylsiloxane)–silica composites, demonstrating that the presence of silica restricted the mobility of the polymer chains.^13^

Nath et al.^14^ used density functional theory (DFT) to describe PDMS oligomers (of 6, 12, or 20 monomers) near the nanosilica surface. The DFT calculations for the density profiles of PDMS atoms near a wall containing oxygen atoms, to represent an amorphous silica surface, were in reasonable agreement with those predicted by fully atomistic molecular dynamics (MD) calculations.^14^ The only visible difference between the DFT and MD density profiles was a slightly higher first peak height of the methyl (CH_3_) density profile in the DFT results, for the smaller PDMS chains (6 and 12 monomers).^14^ A thin film of PDMS oligomers (with 20 monomers) near hydroxylated crystalline and nanosilica was also modeled using classical MD by Tsige et al.^15^ An oscillatory PDMS density profile extending approximately 3 nm from the nanosilica surface was observed, beyond which the density transitioned to that of the bulk region.^15^ The Si–O bond of the backbone was sterically constrained from approaching the surface.^15^ The amplitude of oscillations and the number of peaks were independent of the two types of crystalline (α-quartz, β-crystalline) surfaces and temperature, whereas for amorphous nanosilica, the amplitude was smaller and decayed rapidly.^15^ The methyl end groups had a stronger tendency to accumulate at the surface.^15^ Smith also modeled PDMS oligomers (with 19 monomers) near bare nanosilica and functionalized (with hydroxyl and trimethylsiloxy groups) nanosilica.^16^ It was found that the density of PDMS near the bare silica surface was much greater and the dynamics of interfacial PDMS much slower than that observed for PDMS melts due to a strong van der Waals dispersion interaction.^16^ The presence of hydroxyl and trimethylsiloxy groups on the nanosilica surface resulted in a decrease in the density of interfacial PDMS and a speedup in chain dynamics relative to those observed for PDMS near the bare nanosilica due to increased separation between PDMS and the nanosilica surface and thus a decrease in the strength of the dispersion attraction.^16^ Despite the presence of strong hydrogen bonding interactions between small molecules for hydroxylated nanosilica and PDMS, no significant hydrogen bonding was observed in the classical MD simulations.^16^ Nevertheless, the interaction between hydroxyls and the oxygen atoms of PDMS was found to play a role in the interfacial structure and dynamics of PDMS with low-to-moderate degrees of hydroxylation.^16^

Furthermore, there are other atomistic simulation studies that have investigated the interface of nanosilica with different types of polymers. Bacova et al. found that dynamical heterogeneities existed within the *cis*-1,4-polybutadiene layer, with the chains possessing longer sequences of adsorbed segments (trains) on a nanosilica surface.^17^ Guseva et al. studied a noncross-linked (1,4) *cis*-polyisoprene (PI) melt confined between two amorphous, fully coordinated silica surfaces.^18^ Large-scale atomistic MD simulations were used to model nanosilica in oligomeric polymethyl methacrylate (PMMA)^19^ or polystyrene (PS).^20^ Functionalizing the nanosilica surface by grafting ligands such as hexamethyldisilazane (HMDS) and octyltriethoxysilane (OTES) could improve the adhesion^21^ of polypropylene to the nanosilica and increase the interface strength.^22^ In particular, it was found that OTES functionalization led to the highest adhesion and interfacial strength, resulting in a higher tensile strength.^22^ The structure of PEO chains around the nanosilica interface was investigated by atomistic MD.^23–26^ Hydroxyl end-functionalized PEO chains showed a higher affinity to a hydroxylated nanosilica surface due to hydrogen bonds. The structure of two to three molecular layers was influenced by the nanosilica surface.^24^ Polyethylene glycol (PEG)-adsorbed molecules on the nanosilica surface could not be considered glassy or immobilized since tails and loops on silica showed local mobility.^25^ The adsorbed polymer PEG molecules adopted graft-like conformations, making them extend away from the nanosilica surface to form bridges with other nanosilica particles, which, in turn, drove the formation of a nanoparticle network.^26^ Functionalization with different terminal groups (methoxy or hydroxyl) affected the structure and dynamics of PEG chains.^26,27^ The hydroxyl functionalization played a key role in the formation of the nanosilica network.^26^ The methoxy-functionalization reduced the number of bridges, thereby making the network less dense.^26^ Furthermore, atomistic MD simulations were also used to investigate the static properties of an interface consisting of polyimide–nanosilica nanoparticles with a modified surface.^28,29^ The diffusion of PS chains from a hydroxy (–OH)-terminated silica surface with different grafting densities was also studied based on atomistic MD simulations.^30^

The molecular-level understanding of polymer–nanoparticle interfacial interaction is of great interest for the design of polymer nanocomposites with advanced properties.^32^ Here, we investigate the structure and dynamic properties of neutral and ionic PDMS melts,^33^ for different temperatures, on a nanosilica surface functionalized by either silanols (both isolated and geminal) or silanols with sulfonate groups, by means of atomistic simulations. These systems mimic neutral and ionic PDMS nanosilica composites, respectively.^34^ In particular, we investigate the effect of temperature, PDMS charge localization,^35^ nanosilica surface functionalization, and silica nanoconfinement on the structure and dynamics of PDMS chains (Table 1). The review is organized as follows. Section 2 introduces the applied methodology and simulation details. In Section 3.1, we investigate the structure, and in Section 3.2, the dimensions and conformations of PDMS chains in the interphase region. In Section 3.3, we calculate and compare the diffusion and interfacial properties of PDMS for different ionic polymer architectures, charge densities, and electrostatic strengths. Conclusions are listed in Section 4.

**Table 1:** Static Properties of the Neutral and Functionalized Systems Studied by Means of Atomistic MD at *T* = 375 and 473 K^a^

system	*T*	*R*_g_	*R*	*R*_g_^∥^	*R*_g_^⊥^	*R*_charge_	γ	*N*	number of	*M*_w_	Figure/ref
	[K]	[nm]	[nm]	[nm]	[nm]	[nm]			chains		
•neutral-80	375	2.16	5.40	1.28	1.17	—	—	82	128	6095.7	Figures 3–8
	473	2.19	5.47	1.31	1.17	—	—	82	128	6095.7	Figures 3–8
•neutral-160	375	2.92	6.58	1.77	1.49	—	—	164	64	12176.4	Figures 3–8
	473	2.96	6.97	1.76	1.60	—	—	164	64	12176.4	Figures 3–8
•random-80-copolymer, *f* = 2.4%	375	2.17	5.53	1.20	1.36	8.1	2.0	82	128	6065.7	Figures 3–8
	473	2.22	5.82	1.17	1.49	8.1	1.6	82	128	6065.7	—
•random-80-copolymer, *f* = 10%	375	1.97	4.34	1.03	1.32	2.5	6.6	82	128	6448.4	Figures 3–8
	473	2.10	4.37	1.10	1.41	2.5	5.3	82	128	6448.4	^31^
•random-160-copolymer, *f* = 10%	375	2.42	5.36	1.55	1.01	2.7	6.1	164	64	12881.7	Figures 3–8
	473	2.40	5.27	1.55	0.99	2.7	4.9	164	64	12881.7	^31^
•chain ends, *f* = 2.4%	375	2.68	7.18	1.22	2.06	11.6	1.4	82	128	6183.9	Figures 3–8
	473	2.80	7.55	1.29	2.12	11.6	1.1	82	128	6183.9	^31^

a Radius of gyration *R*_g_, square root of the mean squared end-to-end distance *R*, average charge–charge backbone distance *R*_charge_, Manning number γ, number of monomers *N*, number of chains in the simulation cell, and molecular weight *M*_w_. The excess mass of a N^+^-carrying side chain is 100 g/mol, the mass of a monomer is 74 g/mol, and the mass of the end groups terminating the backbone is 90 g/mol. For three out of the 12 systems, data at 473 K had been obtained from the mentioned reference. All *R* and *R*_g_ values have a statistical error below 4%.

## 2 Methodology

Our nanocomposite systems are composed of neutral or ionic PDMS chains and a nanosilica slab, mimicking PDMS nanocomposites. The ionic PDMS chains carry a permanent positive charge, either on the chain ends or randomly grafted along the backbone (random copolymer) as in the experimental study.^3^ We used ionic PDMS melts with chain charge densities of *f* = 2.5% and *f* = 10%, where *f* is the ratio of functionalized monomers to the total number of monomers, as shown in Figure 1. A united atom model, which does not incorporate hydrogen atoms and is faster than a fully atomistic model, was used to simulate the PDMS melt since its density can be predicted in consensus with experiments.^33,36^ With a monomer length *b*_0_ ≈ 0.26 nm, the contour length is *L* = *Nb*_0_, and the Kuhn length is *b* = *b*_0_ *C*_∞_ ≈ 1.3 with *C*_∞_ = 6*R*_g_^2^/*Nb*_0_^2^ ≈ 5 for an ideal chain, using our values. The reported experimental Kuhn lengths are in the range *b* ∈ [1.1, 1.5] nm^37–39^ for the range of temperatures used in the present study, while *R*_g_ is experimentally known to depend only weakly on temperature, in agreement with our data.

**Figure 1:**
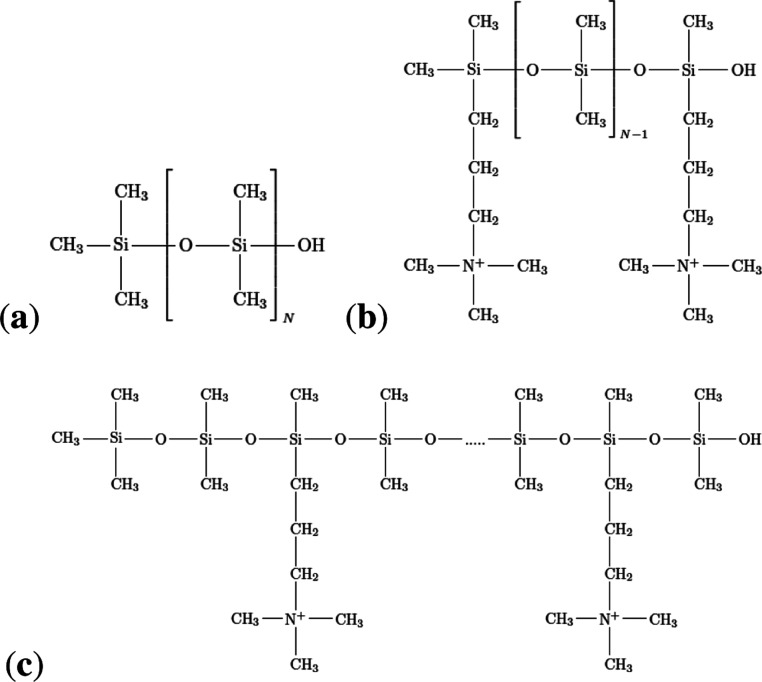
Chemical structures of poly­(dimethylsiloxane). (a) Neutral PDMS chain, (b) cationic PDMS chain functionalized on its chain ends, and (c) functionalized cationic PDMS chain, grafted randomly (random copolymer) along its backbone.

The ionic functionalization of nanosilica was implemented using the -(CH_2_)_3_SO_3_ group (CH_2_ was modeled as a united atom), substituting isolated silanols. The force field for the sulfonate (SO_3_^–^) functionalization of the nanosilica surface was taken from the work by Lin and Maranas.^40^ The nanosilica configuration (of thickness ≈5 nm) and its force field were taken from the work by Geske et al.^41^ Neutral and ionic PDMS melts with polymerization degrees of *N* = 82, 164 were simulated for different temperatures and charge localizations and subjected to periodic boundary conditions. To account for PDMS chain polarizability, we scaled down the charges of nitrogen (N^+^) by 50%.^42^ A realistic amorphous nanosilica slab was used^41^ to form the PDMS/nanosilica interphase, as shown in Figure 2. To compensate for the charge of the PDMS chains for the charge density of *f* = 2.4%, the nanosilica surface was functionalized with 191 sulfonate (SO_3_^–^) groups, while for *f* = 10%, it was functionalized with 798 sulfonate (SO_3_^–^) groups. In experimental ionic-PDMS–silica nanocomposites, the Br^–^ counterions have been removed (as in our work); however, a 1:1 ratio between positive and negative charges was not possible to achieve.

**Figure 2:**
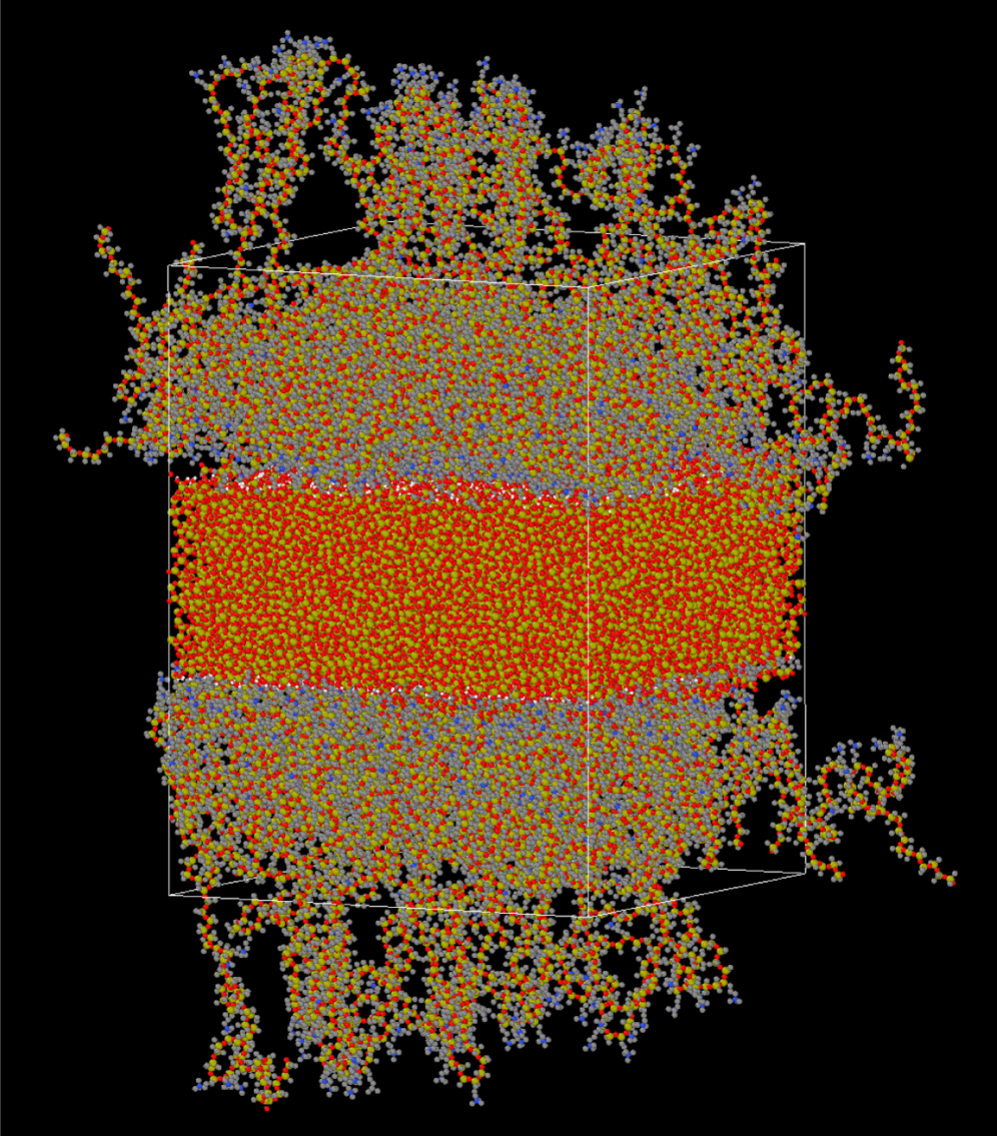
PDMS–nanosilica interphase-containing silanols. The number of monomers per chain is *N* = 82, and the chains were randomly grafted (random copolymer). Atom colors: H (light blue), C (gray), N (blue), O (red), and Si (light green). The thin white line marks the simulation box edges.

The Lorentz–Berthelot mixing rules $\epsilon_{ij}={\left(\epsilon_{i}\epsilon_{j}\right)}^{1/2}$ and σ_ij_ = (σ_i_ + σ_j_)/2 were used to model the interaction between PDMS and nanosilica surfaces.^43^ In addition, the Coulomb interaction between charged united atoms was incorporated and given by

1 $$V_{ij}^{Coulomb}=\frac{q_{i}q_{j}}{4\pi \epsilon_{r}\epsilon_{0}r_{ij}},$$

where *q*_*i*_ is the charge of atom *i* and *r*_*ij*_ is the Euclidean center–center distance between atoms *i* and *j*. The long-range electrostatics were computed using the particle-mesh Ewald (PME) method, and the real-space cutoff was 14.5 Å. The van der Waals forces were calculated up to 14.5 Å.^36^ The system was first energy-minimized using a steep integrator.^44^ We ensured that the linear size of the simulation cell was larger than the root-mean-square end-to-end distance of the polymer chains. Subsequently, MD simulations were performed using the isothermal isochoric ensemble (NVT) since it is more efficient at temperatures of 473 and 375 K. The dimensions of the simulation cell are *L*_*x*_ = *L*_*y*_ = 12.1632 nm and *L*_*z*_ = 16.3218 nm. The simulation cell contains more than 100,000 atoms.

In order to integrate Newton’s equation of motion, the leapfrog algorithm was used.^44^ The temperature *T* was kept constant using a Nosé–Hoover thermostat with a relaxation time of 2 ps. Integration time steps equal to Δ*t* = 0.5 fs were used for the ionic PDMS/silica mixtures, while Δ*t* = 0.8 fs was used for the neutral PDMS/silica nanocomposite systems. The MD simulations were performed using the GROMACS package with GPU support.^44–47^

## 3 Results and Discussion

We focus our attention on the neutral and ionic (cationic) PDMS chain structure, dimensions, conformations, and dynamics (diffusion) of all systems studied (Table 1) by atomistic MD simulations.

### 3.1 Structure

First, the probability density of PDMS chains (neutral or ionic), calculated as a function of the distance from the nanosilica surface is depicted in Figure 3. As can be seen, there is stronger layering at lower temperature (375 K) and for the case of ionic PDMS chains (Figure 3e–h) around the anionic nanosilica surface than for neutral PDMS/surfaces (Figure 3a–d). The probability density of neutral PDMS chains with *N* = 82 monomers does not show any layering and extends up to ≈7 nm from the nanosilica surface. Beyond this, a depletion layer is created, while the chains with *N* = 164 extend along the whole *z* direction in the simulation cell, as can be seen in Figure S1. However, the smallest depletion, adjacent to the surface, is depicted for the ionic PDMS chains, as shown in Figure 3. In particular, for ionic PDMS chains with *N* = 82 and *f* = 2.4%, the depletion layer appears at 5 nm, while for *f* = 10%, it is ≈4 nm away from the nanosilica surface. In addition, the layering of ionic PDMS chains on the nanosilica surface is stronger than that for the neutral PDMS/nanosilica mixtures. The density of PDMS (with *N* = 80), far from the nanosilica slab, was ≈0.783 g/cm^3^ at 473 K (Figure S1), whereas a value of 0.825 g/cm^3^ was reported for high *M*_w_ PDMS liquids at 473 K.^48^

**Figure 3:**
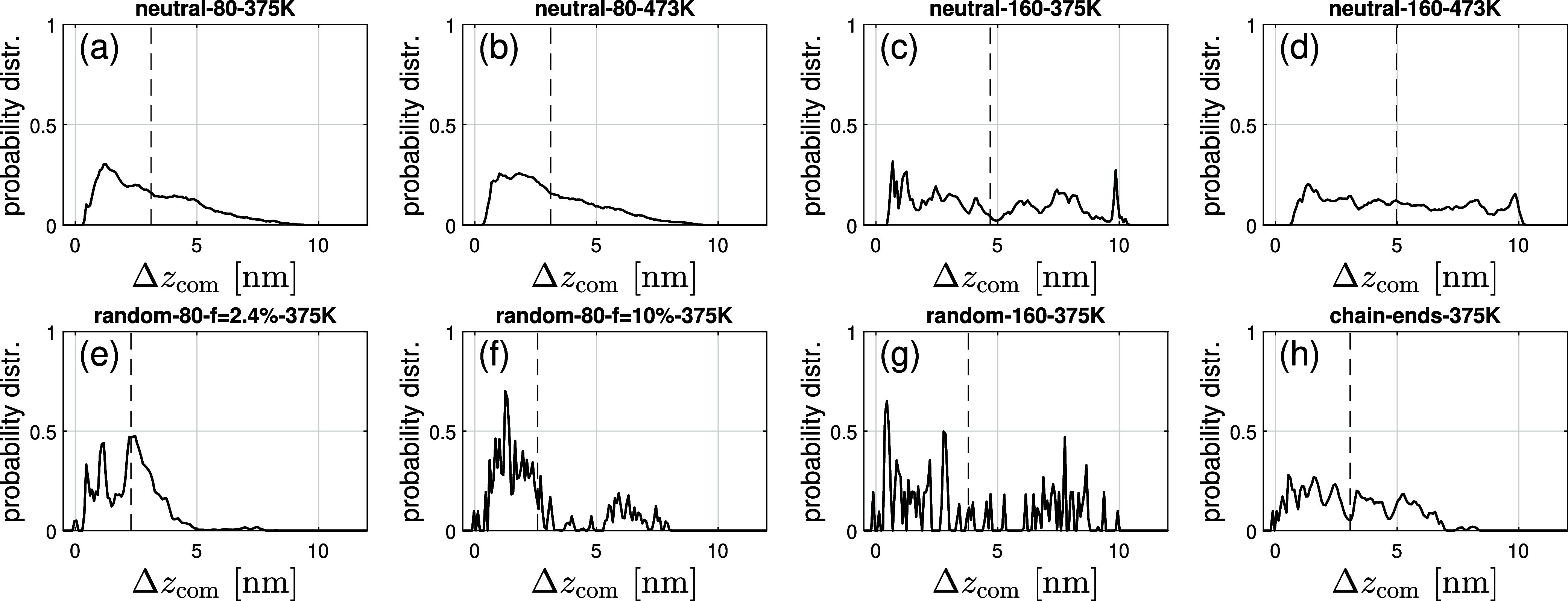
Probability distribution of the chain’s center of mass position versus the distance Δ*z*_com_ from the Si surface for eight selected systems from Table 1: (a) neutral system with *N* = 82 at 375 K, (b) neutral system with *N* = 82 at 473 K, (c) neutral system with *N* = 164 at 375 K, (d) neutral system with *N* = 164 at 473 K, (e) random *f* = 2.4% copolymer with *N* = 82 at 375 K, (f) random *f* = 10% copolymer with *N* = 82 at 375 K, (g) random copolymer with *N* = 164 at 375 K, and (h) ionic chains functionalized on chain ends with *N* = 82 at 375 K. The dashed vertical and horizontal lines mark the averages.

Second, the radial distribution function (RDF) or pair correlation function *g*_AB_(r)^44,49^ between different types of atoms is calculated, providing the local spatial ordering in the isotropic melt, where *V* is the total volume of the system and *N*_A_ and *N*_B_ are the numbers of type A and B atoms, respectively. We calculate the RDF between the H and O atoms, shown for both temperatures in Figure 4a,b. As can be seen in these figures, there is a stronger correlation between H atoms of hydroxyls and O atoms of PDMS in the neutral PDMS/nanosilica interface than in the ionic random copolymer PDMS/nanosilica interface. In addition, for the ionic chain-end PDMS, there is a much stronger correlation between H atoms of the hydroxyls and O of SO_3_^–^ due to the stronger electrostatic interaction present. Temperature has a negligible effect on the H–O RDF of neutral PDMS chains, showing a weaker correlation (Figure 4a,b). However, a much weaker correlation with electrostatic strength appears for the ionic chain-end PDMS chains. A similar picture appears in the RDF between the H atoms of hydroxyl groups and the Si atoms of PDMS. Hydrogen bonding between the hydroxyls (-OH, donors) of silanols (both isolated and geminal) and -*O*- (acceptor) of the PDMS backbone may be formed.^50,51^ To determine if a hydrogen bond exists, a geometrical criterion is used, with the OH^–^··O distance and H–O–O angle below 0.35 nm and 30°. We depict the number of hydrogen bonds with charge density, for all the systems studied in Figure 5. It can be seen that hydrogen bonds between silanols and oxygen of PDMS are affected by the molecular weight of neutral PDMS chains, denoting strong hydrogen bonding for neutral PDMS chains (with *N* = 164), in agreement with experiments.^52^ Temperature decreases the number of hydrogen bonds for the neutral PDMS and ionic chain-end PDMS, whereas it has no effect on the other ionic systems. The chain-end ionic PDMS chains have a higher number of hydrogen bonds in comparison to the ionic random PDMS copolymers (for both *f* = 2.4% and *f* = 10%).

**Figure 4:**
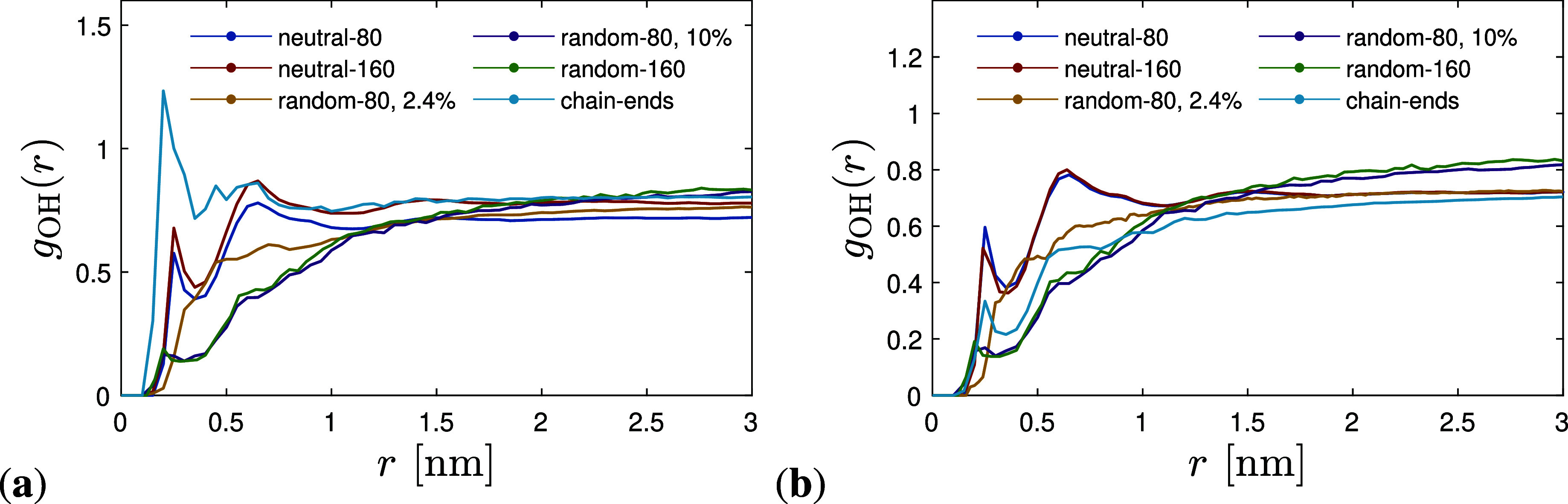
Radial O–H pair correlation function *g*_OH_(*r*) versus O–H distance *r* at (a) *T* = 375 K and (b) *T* = 473 K for all systems studied.

**Figure 5:**
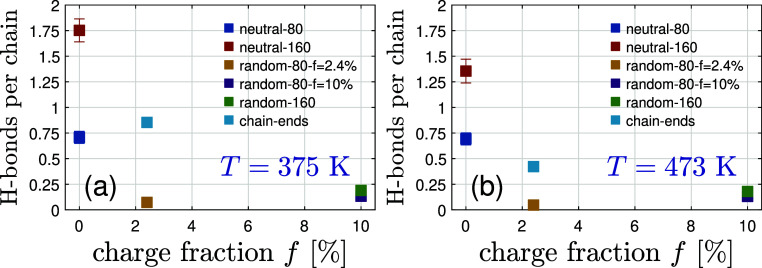
Time-averaged number of H-bonds per chain vs charge fraction for all systems from Table 1 at (a) *T* = 375 K and (b) *T* = 473 K.

The electrostatic strength of these two types of charge-sequenced polymers can be quantified by eq 2. This dimensionless ratio is known as the Manning number, which we adopt here from the field of polyelectrolyte solutions.^53^

2 $$\gamma =\frac{\lambda_{B}}{R_{charge}}$$

between the Bjerrum length, λ_B_, and the average distance, *R*_charge_, along the backbone of the charged monomers on the axis of the fully extended ionic PDMS chain, *R*_charge_ (the backbone length distance between Si atoms, in a PDMS chain, is 0.29 nm). The Bjerrum length given by eq 3 is varied in this work, through temperature changes.^54^

3 $$\lambda_{B}=\frac{e^{2}}{\varepsilon_{r}k_{B}T},$$

where *e* is the elementary charge, ϵ_*r*_ is the permittivity of the medium, *k*_B_ is the Boltzmann constant, and *T* is the temperature of the system. Using ε_*r*_ = 2.7 for PDMS, the λ_B_ decreases from 16.6 to 13.1 nm between *T* = 375 K and *T* = 473 K.

### 3.2 Chain Conformations

Furthermore, we focused our attention on the analysis of the dimensions and conformations of all PDMS systems. The radius of gyration (*R*_g_) of a molecule is defined as the square root of the mean squared distance between the monomers and the center of mass of the chain and is thus given by^44,55^

4 $$R_{g}^{2}\left(N\right)=\frac{1}{\sum_{i=1}^{N}m_{i}}\left\langle \sum_{i=1}^{N}m_{i}∥r_{i}-r_{cm}{∥}^{2}\right\rangle ,$$

where r_*i*_ is the position of atom *i*, *m*_*i*_ is its mass, and r_cm_ is the center of mass of the chain. The average is taken across all chains.

Specifically, Figure 6 depicts the *R*_g_ spatial distribution (as a function of distance from the nanosilica surface) of neutral and ionic PDMS chains, respectively, for different *M*_w_ values and charge localization. The ionic chain-end-functionalized polymers are stretched more than neutral or ionic random copolymers. The ionic chain-end PDMS chains appear to have a stronger interaction, with the SO_3_^–^-functionalized nanosilica surface, through the cationic ammonium N^+^, than the ionic random PDMS copolymers,^31^ which have higher mobility, allowing them to rearrange and expand their conformations. A similar behavior, with a stronger interaction between cationic ammonium N^+^ and Br^–^ counterions, and a stretched conformation was also observed in ionic PDMS melts.^33^ The conformations of ionic random PDMS copolymers, at higher electrostatic strength, γ (or *f* = 10%), are more collapsed in comparison with neutral PDMS chains, as can be seen from the *R*_g_ values of Table 1 and Figure 6. Temperature has a very weak effect on both *R*_g_ and end-to-end distance (*R*) for neutral and ionic random copolymers, while for the ionic chain-end PDMS polymers, *R*_g_ and *R* tend to rise with increasing temperature (Table 1). By increasing the temperature, the electrostatic strength (Manning number) decreases, indicating weaker electrostatic interactions, which allows the ionic PDMS chains expand, particularly for ionic random copolymers (Table 1). The *R*_g_ spatial distribution is rather homogeneous for neutral PDMS with *N* = 82 monomers, while for neutral PDMS with *N* = 164 monomers, higher *R*_g_ values are observed 5 nm away from the nanosilica surface. Homogeneous *R*_g_ spatial distributions are also observed for the ionic random copolymer PDMS. However, for the ionic chain-end PDMS, the *R*_g_ increases with distance from the nanosilica surface.

**Figure 6:**
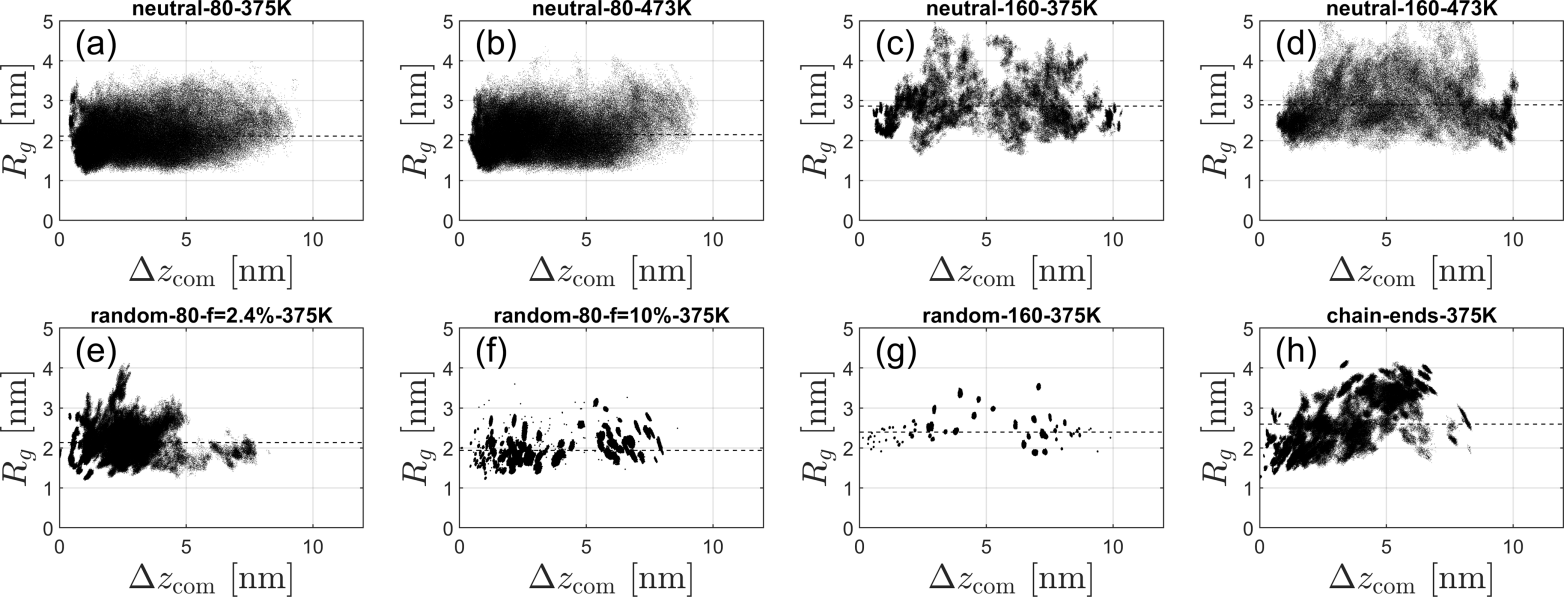
Radius of gyration *R*_g_ for each chain separately, averaged over time as a function of its center of mass altitude Δ*z*_com_, for the same eight systems selected for Figure 3. Each chain gives rise to a black dot. The number of dots is not smaller in panels (f, g) compared with other panels. For these systems, the number of visible dots is smaller as the dimensions of the PDMS chains and their distance to the surface change very little during the time span of 300 ns, which is also reflected by Figure 3g.

Beyond the overall radii of gyration, we calculated the *R*_g_ spatial distribution (as a function of distance from the nanosilica surface) of all individual chains, as well as the eigenvalues (diagonal components) *G*_*xx*_, *G*_*yy*_, and *G*_*zz*_ of the tensors of gyration of all individual chains and their center-of-mass positions, and extracted various shape parameters, in order to further investigate the conformation of PDMS chains in the interphase, as shown in Figures 7 and 8. The *R*_g_ is related to the diagonal components by the following equation

5 $$R_{g}^{2}={\left(R_{g}^{⊥}\right)}^{2}+2{\left(R_{g}^{∥}\right)}^{2}=G_{xx}+G_{yy}+G_{zz}$$

The perpendicular and the lateral components of the gyration tensor are

6 $$R_{g}^{⊥}=\sqrt{G_{zz}},,R_{g}^{∥}=\sqrt{\left(G_{xx}+G_{yy}\right)/2}$$

**Figure 7:**
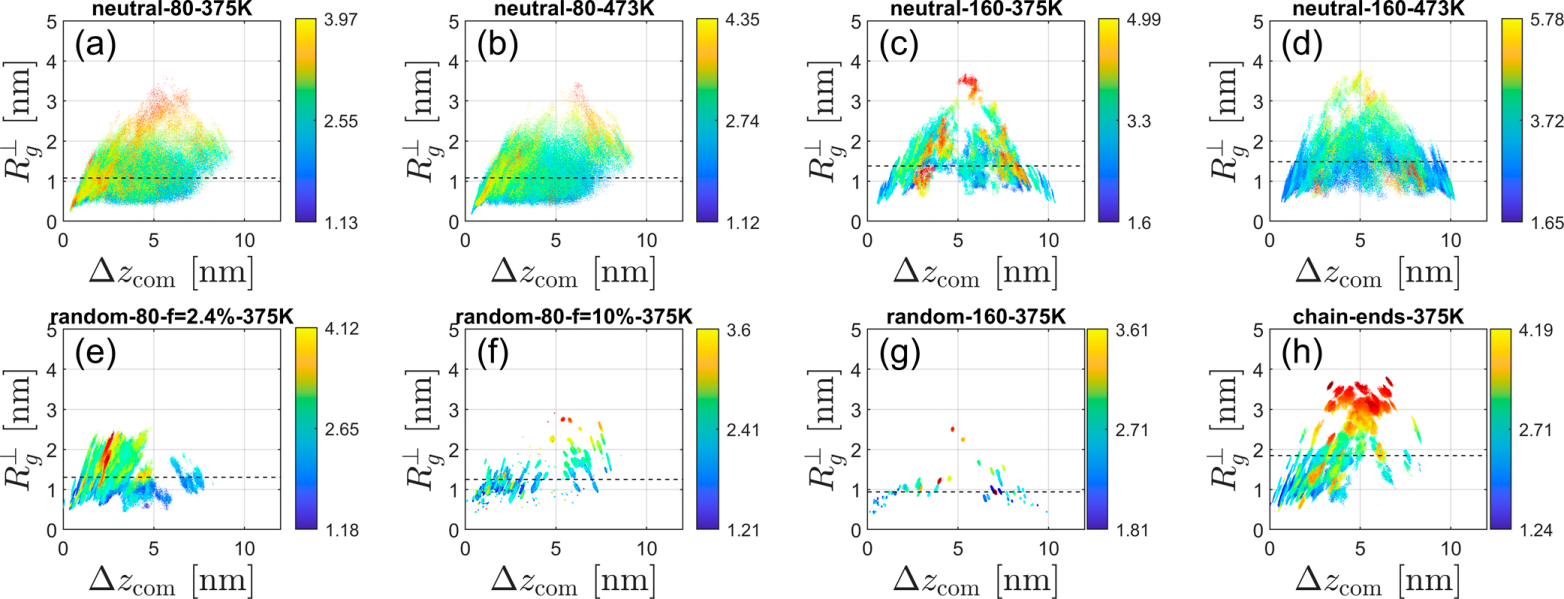
Perpendicular component of the gyration tensor, $R_{g}^{⊥}=\sqrt{G_{zz}}$ , for each chain separately as a function of its center of mass altitude Δ*z*_com_, for the same eight systems selected for Figure 3. Each chain gives rise to a colored dot, where the color encodes the *R*_g_ value for this chain (see the color bar).

**Figure 8:**
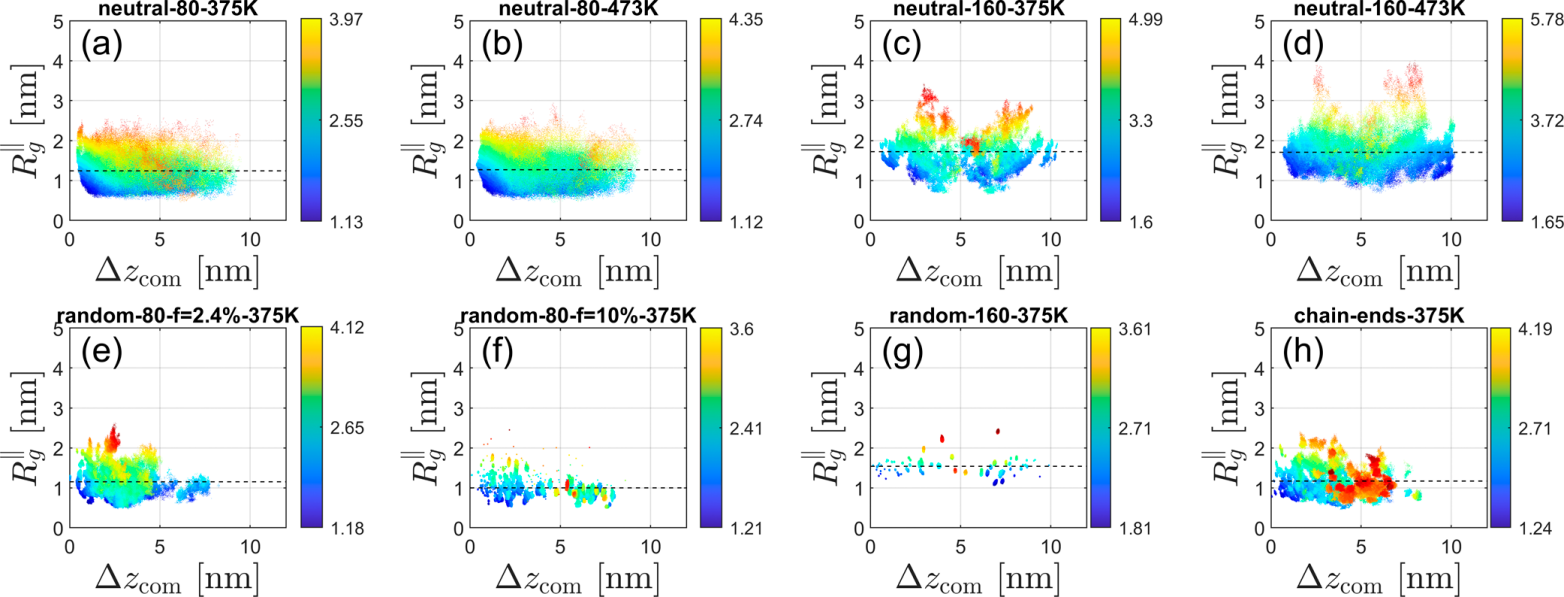
Lateral component of the gyration tensor, $R_{g}^{∥}=\sqrt{\left(G_{xx}+G_{yy}\right)/2}$ , for each chain separately as a function of its center of mass altitude Δ*z*_com_, for the same eight systems selected for Figure 3. Each chain gives rise to a colored dot, where the color encodes the *R*_g_ value for this chain (see the color bar).

For ionic chain-end PDMS specifically, both the perpendicular and lateral components of the gyration tensor are more stretched as the distance from the nanosilica surface increases, as shown in Figures 7h and 8h. For ionic random copolymers and neutral chains, the perpendicular and lateral components exhibit a homogeneous distribution, with a broader distribution for the neutral PDMS chains.

### 3.3 Chain Dynamics

In this section, we discuss the measured diffusion coefficients and dynamics of all systems studied (Table 2), obtained by MD, from the asymptotic behavior of the mean square displacement (MSD),^56,57^ of all atoms in a PDMS chain, given by the equation

7 $$D_{0}=\frac{1}{6}\lim_{t\to \infty }\frac{d}{dt}\left\langle |r_{i}\left(t\right)-r_{i}\left(0\right){|}^{2}\right\rangle ,$$

where ⟨|r_*i*_(*t*) – r_*i*_(0)|^2^⟩ is the time-dependent MSD of the particles (atoms) of chains, averaged over time and over the atoms of the ensemble. We extrapolated the *D*_0_ by fitting eq 7 to the simulation data at long times, as in the Kremer–Grest model,^58^ of the systems which appear to be in a subdiffusive regime, when the effective minimal slope (β) at long times is 0.5 < β ≤ 0.85.

**Table 2:** Diffusion Coefficients *D*_0_ of the PDMS Atoms, Calculated Using Eq 7, at Long Simulation Times, for Different Systems and Temperatures Studied, and Effective Minimal Slopes β

system	*T* [K]	*D*_0_ [pm^2^/ps]	β
•neutral-80	375	11.0	1
	473	20.8	0.85
•neutral-160	375	3.3	0.80
	473	8.8	0.70
•random-80 copolymer, *f* = 2.4%	375	-	0.50
	473	2.9	0.55
•random-80 copolymer, *f* = 10%	375	-	0.36
•random-160 copolymer, *f* = 10%	375	-	0.14
•chain ends, *f* = 2.4%	375	-	0.60

It is noted that the ionic charges on the polymer chains hinder the PDMS chain dynamics (and thus reduce their diffusion) as shown in Figure 9a and Table 2, which was also observed by coarse-grained simulations of ionic nanocomposites.^59^ The chains in the neutral PDMS/nanosilica interphase diffuse faster at all temperatures, whereas the ionic chain-end PDMS appears to have much slower dynamics due to stronger electrostatic attraction and more contacts with the ionically functionalized nanosilica, as can be observed in Figure 9a. The charge density *f*, or electrostatic strength decreases dramatically in the dynamics of ionic random PDMS copolymers, with those PDMS chains having *f* = 10% being immobilized near the nanosilica surface. The ionic chain-end PDMS chains appear to have a lower diffusion than the ionic random PDMS copolymers (*f* = 2.4%) due to stronger electrostatic interaction, with the SO_3_^–^-functionalized nanosilica surface and a higher number of hydrogen bonds (Figure 5).

**Figure 9:**
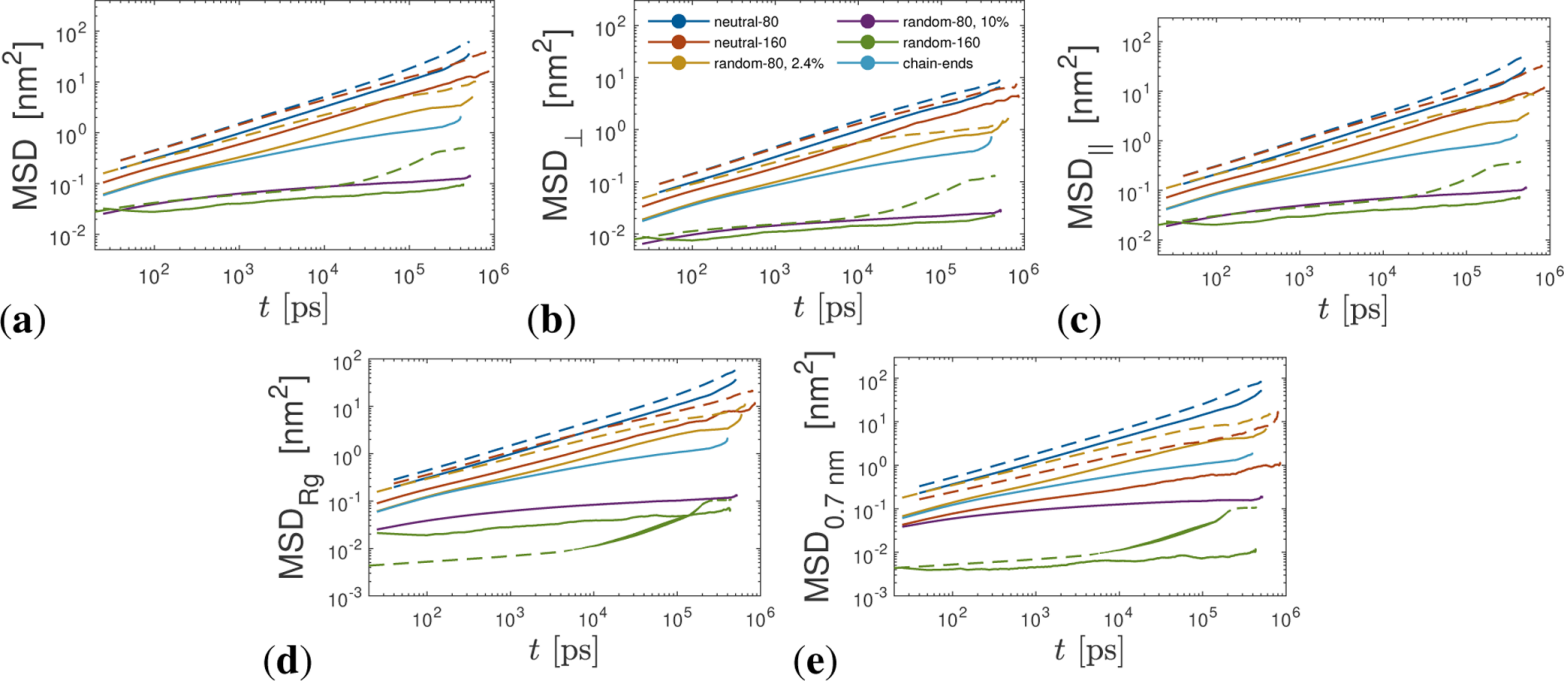
Various mean square displacement measures at *T* = 375 K (solid lines) and *T* = 473 K (dashed lines) versus time *t* for all systems studied. (a) MSD, (b) MSD_⊥_, (c) MSD_∥_, (d) MSD_R_g__, and (e) MSD_0.7nm_ data. The legend in panel (b) applies to all panels.

Furthermore, we calculated the MSD of the ionic PDMS chains perpendicular to the surface (MSD_⊥_) (Figure 9b) and parallel to the surface (MSD_∥_) (Figure 9c). We found anisotropic dynamical PDMS behavior for all systems (both neutral and ionic) studied, where the dynamics parallel to the surface were faster than those of their perpendicular counterpart. The largest anisotropy appeared at *T* = 473 K for the longest (*N* = 164) ionic PDMS chains, as can be seen in Figure S2. In our previous work,^31^ it was observed that the dynamics of short ionic random PDMS copolymers (*N* = 42) were nearly isotropic. The ionic PDMS chains are not desorbed from the nanosilica surface, whereas the neutral PDMS (with *N* = 80) chains, which are the most mobile, desorb at a rate of ≈0.02–0.05 ns^–1^.

Furthermore, it was observed by dielectric spectroscopy in attractive P2VP/nanosilica composites that the interfacial P2VP/nanosilica layer had suppressed segmental dynamics and that the suppression increased with decreasing polymer *M*_w_. In order to investigate the interfacial dynamics, in our systems, we calculated the MSD of neutral and ionic PDMS chains whose center of masses resided, at *t* = 0, inside an *R*_g_ distance from the nanosilica surface and have depicted them in Figure 9d. It can be observed that for ionic PDMS chains with *f* = 10%, the chain dynamics is dramatically suppressed inside the *R*_g_ region from the nanosilica surface, as observed in attractive nanocomposites.^60–62^ The PDMS dynamics from the nanosilica surface appears heterogeneous, with dynamics faster away from the nanosilica surface, for both neutral and ionic PDMS, as can be seen in Figure S2. In particular, for the charge density *f* = 10% and at *T* = 375 K, ionic PDMS chains (*N* = 82, 164) cannot desorb from the *R*_g_ region to the bulk region at *T* = 375 K (whereas at *T* = 473 K, only one chain desorbs to the bulk region); thus, those ionic PDMS chains remain bound. This bound layer thickness could possibly be tuned by changing the nanosilica size.^12^ For the ionic PDMS systems, a few chains can desorb from *R*_g_ to the bulk region. For ionic random PDMS chains with *f* = 10%, slower dynamics is observed at times up to 10 ns, inside the *R*_g_ region, whereas the bulk reptation dynamics is recovered at sufficiently long times (Figure 9d). A similar result is observed, for chain dynamics, by coarse-grained MD simulations, for the unadsorbed chains near a nanofiller.^63^

In addition, we also calculated the MSD of PDMS chains whose centers of mass are initially at *t* = 0, within a close distance (less than 0.7 nm) from the nanosilica surface, as depicted in Figure 9e. It was observed that the neutral PDMS chains exhibited much faster dynamics than the ionic PDMS chains, near the nanosilica surface, since the ionic attractive interaction (between the N^+^ of PDMS and SO_3_^–^ of nanosilica) is stronger than the van der Waals dispersion and hydrogen bonding interactions.^64^ Furthermore, the ionic random copolymers and ionic chain-end PDMS can be considered immobile at *T* = 375 K (the ionic random PDMS chains with *N* = 164 are immobile for both *T* = 375, 473 K), due to their strong adhesion with nanosilica,^65^ whereas neutral PDMS chains remain mobile, as can be seen in Figure 9e.

## 4 Conclusions

In summary, we investigated the structure, conformations, and dynamics (diffusion) of neutral and ionic PDMS (with cationic ammonium randomly grafted along the backbone or on the chain ends) melts, on the interface with nanosilica (functionalized with either hydroxyl or SO_3_^–^), using atomistic MD simulations. The type of interaction at the interface alters both the structural and the dynamic behavior. A stronger layering structure was observed for the ionic random PDMS copolymers, which are contracted in comparison with the neutral PDMS chains. However, neutral PDMS chains diffused faster due to the van der Waals dispersion and hydrogen bonding interactions, which are weaker than electrostatic interactions. Substantial hydrogen bonding exists for neutral PDMS, and it decreases with temperature and polymer *M*_w_. Electrostatic strength for ionic random copolymers could dramatically alter chain conformations and dynamics (diffusion). Neutral PDMS (*N* = 160) and ionic PDMS with a low Manning number (γ) appear to have a subdiffusive behavior. We also found that the chemical location of the charge affected both the structural and dynamic behavior. Although ionic chain-end PDMS chains had a low γ, denoting a low electrostatic strength, they had more stretched conformations (*R*_g_) and slower dynamics than the ionic random copolymer PDMS (with *f* = 2.4%), due to the stronger interaction of the cationic ammonium group with the oppositely charged nanosilica surface and a higher number of hydrogen bonds.

Anisotropic dynamics was observed perpendicular and parallel to the nanosilica surface for all of the PDMS polymers studied. The ionic random PMDS copolymers with a high Manning ratio (or *f* = 10%) appeared to have negligible mobility (immobile chains) in the vicinity of the nanosilica surface, having been strongly adsorbed due to their charge fraction, leading to the highest adhesion. The charge density, electrostatic strength, or *M*_w_ significantly decreased the PDMS interfacial dynamics.

## Supplementary Material



## Notes

All data presented in figures, as well as a full trajectory file are available at 10.3929/ethz-b-000726334
Additional MSD-type data (PDF)
