# Supplementary material for: Structure, Conformations, and Diffusion in PDMS/Silica Nanocomposites via Atomistic MD Simulations
Source: Macromolecules. 2025 Nov 25;58(23):12429–39. doi: 10.1021/acs.macromol.5c01745 (PMC12874640; doi:10.1021/acs.macromol.5c01745)
Supplement: Supplementary file 1 [file ma5c01745_si_001.pdf]

SUPPLEMENTARY INFORMATION

Structure, conformations and diffusion in PDMS/silica nanocomposites via atomistic MD simulations

Argyrios V. Karatrantos,[a,*], Nigel Clarke,[b], Lyazid Bouhala,[a] Clement Mugemana,[a] and Martin Kröger [c,d]

[a] *Materials Research and Technology, Luxembourg Institute of Science and Technology, 5, Avenue des Hauts-Fourneaux, L-4362 Esch-sur-Alzette, Luxembourg.*

[b] *School of Mathematical and Physical Sciences, University of Sheffield, 5, Sheffield, S3 7RH, UK.*

[c] *Computational Polymer Physics, Department of Materials, ETH Zurich, 8093 Zurich, Switzerland.*

[d] *Magnetism and Interface Physics, Department of Materials, ETH Zurich, 8093 Zurich, Switzerland.*

∗ *Corresponding author. E-mail: argyrios.karatrantos@list.lu (A.V.K.)*

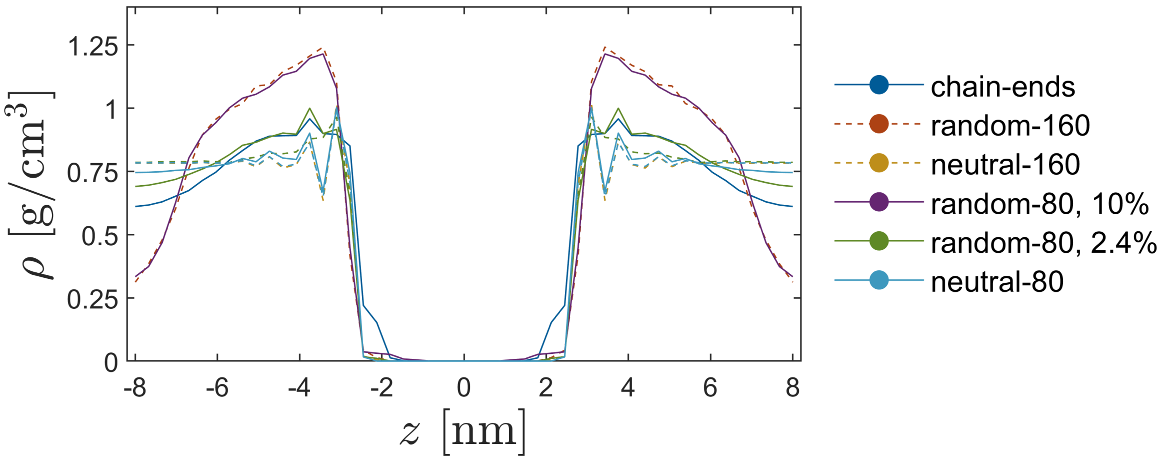

Figure S1: Density ρ versus coordinate z, while $z = 0$ is located in the center of the nanosilica, for selected systems. Solid lines are for $T = 375$ K, dashed lines for $T = 473$ K.

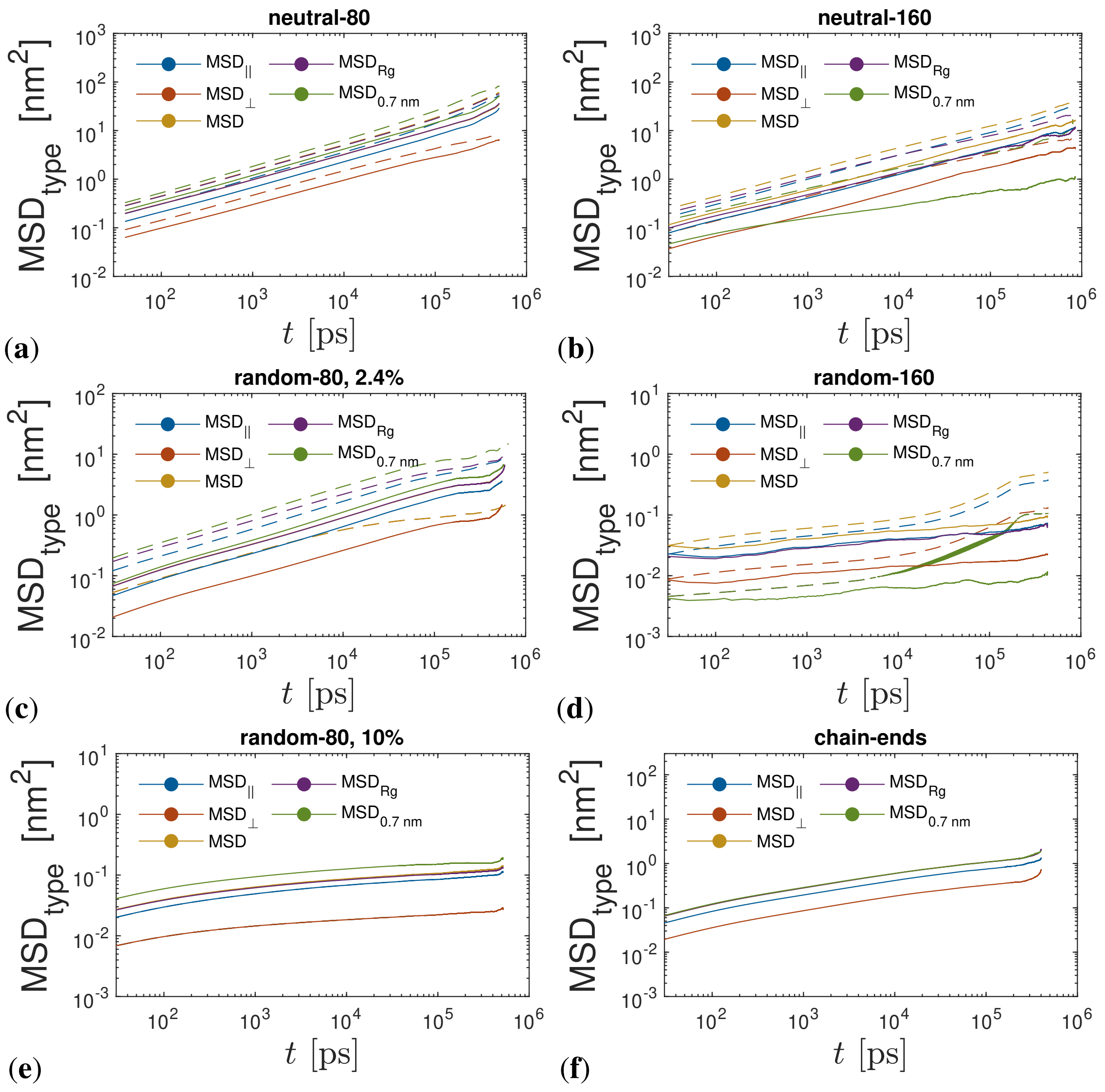

Figure S2: All MSD-type data for (a) the neutral-80, (b) neutral-160, (c) random-80, 2.4%, (d) random-160, (e) random-80, 10%, and (f) chain-ends at $T = 375$ K (solid lines) and $T = 473$ K (dashed lines, if available).
